# Modelling hepatitis D virus RNA and HBsAg dynamics during nucleic acid polymer monotherapy suggest rapid turnover of HBsAg

**DOI:** 10.1038/s41598-020-64122-0

**Published:** 2020-05-12

**Authors:** Louis Shekhtman, Scott J. Cotler, Leeor Hershkovich, Susan L. Uprichard, Michel Bazinet, Victor Pantea, Valentin Cebotarescu, Lilia Cojuhari, Pavlina Jimbei, Adalbert Krawczyk, Ulf Dittmer, Andrew Vaillant, Harel Dahari

**Affiliations:** 1 The Program for Experimental & Theoretical Modeling, Division of Hepatology, Department of Medicine, Stritch School of Medicine, Loyola University Medical Center, Maywood, IL USA; 2 Network Science Institute, Northeastern University, Boston, MA USA; 3 Replicor Inc., 6100 Royalmount Avenue, Montreal, Quebec H4P 2R2 Canada; 4 Department of Infectious Diseases, Nicolae, Testemiţanu State University of Medicine and Pharmacy, Chișinău, Moldova; 5 Toma Ciorbă Infectious Clinical Hospital, Chișinău, Moldova; 6 Institute for Virology, University Hospital Essen, University of Duisburg-Essen, Essen, Germany; 7 Department of Infectious Diseases, University Hospital Essen, University of Duisburg-Essen, Essen, Germany

**Keywords:** Infectious diseases, Viral hepatitis

## Abstract

Hepatitis D virus (HDV) requires hepatitis B surface antigen (HBsAg) for its assembly and release. Current HBV treatments are only marginally effective against HDV because they fail to inhibit HBsAg production/secretion. However, monotherapy with the nucleic acid polymer REP 2139-Ca is accompanied by rapid declines in both HBsAg and HDV RNA. We used mathematical modeling to estimate HDV-HBsAg-host parameters and to elucidate the mode of action and efficacy of REP 2139-Ca against HDV in 12 treatment-naive HBV/HDV co-infected patients. The model accurately reproduced the observed decline of HBsAg and HDV, which was simultaneous. Median serum HBsAg half-life (t_1/2_) was estimated as 1.3 [0.9–1.8] days corresponding to a pretreatment production and clearance of ~10^8^ [10^7.7^–10^8.3^] IU/day. The HDV-infected cell loss was estimated to be 0.052 [0.035–0.074] days^−1^ corresponding to an infected cell t_1/2_ = 13.3 days. The efficacy of blocking HBsAg and HDV production were 98.2 [94.5–99.9]% and 99.7 [96.0–99.8]%, respectively. In conclusion, both HBsAg production and HDV replication are effectively inhibited by REP 2139-Ca. Modeling HBsAg kinetics during REP 2139-Ca monotherapy indicates a short HBsAg half-life (1.3 days) suggesting a rapid turnover of HBsAg in HBV/HDV co-infection.

## Introduction

Chronic hepatitis B virus (HBV) and hepatitis D virus (HDV) co-infection affects an estimated 15–40 million persons worldwide^1,2^ and is the most aggressive form of viral hepatitis^3^. Therapy with pegylated interferon-α2a (pegIFN) is suboptimal in controlling HDV infection^4,5^ and no other therapies are approved for the treatment of HDV.

HDV requires hepatitis B surface antigen (HBsAg) for assembly and release. While the large isoform (L-HBsAg) is not requisite for HDV assembly and release, it is necessary for infectivity^6^. Drugs that directly target HDV and reduce HDV levels are in development^7^, however the only anti-HBV treatment that affects HBsAg production is the nucleic acid polymer (NAP) REP 2139-Ca, which is accompanied by declines in both HBsAg and HDV RNA^8–11^. Therefore, analyzing antiviral response during REP 2139-Ca monotherapy provides a unique opportunity to examine HBsAg production and clearance rates in HBV/HDV co-infected patients and to obtain a deeper understanding of REP 2139-Ca mode of action and efficacy against HDV.

The aim of this study was to analyze the kinetics of HBV DNA, HBsAg, ALT and HDV RNA during REP 2139-Ca monotherapy and investigate the dynamics of HDV RNA and HBsAg using mathematical modelling.

## Results

### Viral-host kinetics during REP 2139-Ca monotherapy

**Figure 1:**
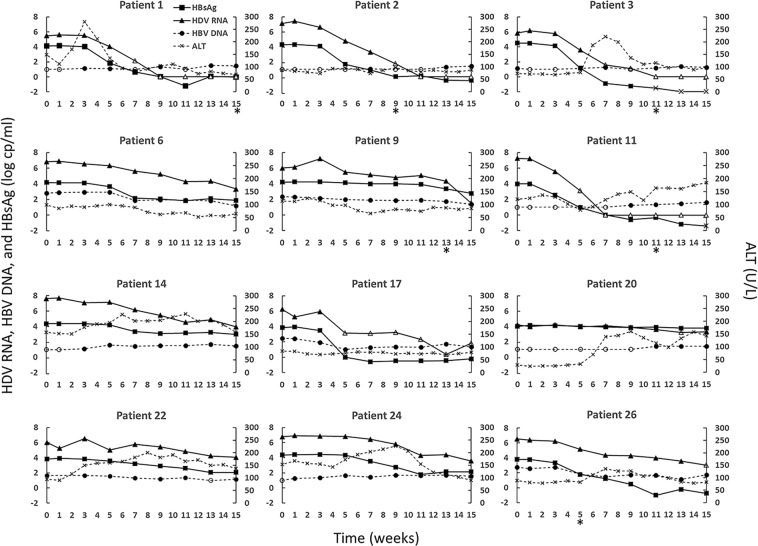
HDV RNA (triangles), HBV DNA (circles), HBsAg (squares), and ALT (x) kinetics during 15-week REP 2139-Ca monotherapy. Point markers with no fill indicate a measurement below the LLoQ, markers with gray fill indicate TND, and an asterisk below a week number indicates the point at which the given patient’s level of Anti-HBs surpassed the LLoQ (i.e., 10 mIU/mL) if at all. LLoQ is HBsAg: 0.05 IU/mL, HDV RNA: 1800 U/mL, HBV DNA: 10 IU/mL.

The kinetics of HBV DNA, anti-HBs, ALT, HBsAg and HDV for individual participants are described in Fig. 1. The pre-treatment HBV DNA was <3 log_10_ IU/mL in all 12 participants and it was <LLoQ in 5 cases (Table 1). HBV DNA levels varied by <1 log_10_ over the course of treatment and did not correlate with observed responses in HBsAg and HDV RNA levels.

Pre-treatment ALT had a median value of 97 U/L [interquartile range:85.5–128]. Half of the cases experienced transient ALT increases and the highest ALT flare was 281 U/L. The median pre-treatment HBsAg titer was 4.15 [3.96–4.31] log_10_ IU/mL. Two patients (Pt 20 and Pt 22) experienced HBsAg decline <1 log_10_ IU/mL from baseline and were classified as non-responders. A third patient, Pt 9, was a borderline responder, who had a minimal HBsAg decrease and HDV decreased only towards the end of treatment. Nonetheless, due to the magnitude of HDV decline, we chose to include Pt 9 in our modeling analysis. After a delay of 3–7 weeks during which HBsAg remained at pre-treatment levels, all responding patients exhibited a biphasic decrease in HBsAg, except for Pt 9 who had a monophasic HBsAg decline (Fig. 1). The median anti-HBs titers at week 15 post initiation of monotherapy in the 6 patients who experienced seroconversion (Fig. 1) was 25 [IQR: 23–30] mIU/mL.

The median pre-treatment HDV level was 6.7 [6.1–7.1] log_10_ U/mL. In the 2 HBsAg non-responders, HDV decline was either 0.83 log_10_ U/mL from baseline (Pt20) or transiently increased but eventually declined to 2.48 (Pt22) log_10_ U/mL from baseline. After a 3–7 week delay during which HDV remained at pre-treatment levels, 5 of 10 responding patients experienced a rapid monophasic decline in HDV either reaching LLoQ or TND and 5 had a biphasic pattern with a slower 2^nd^ phase HDV decline (Fig. 1). By the end of therapy, the 10 responding patients experienced median HDV declines of 4.4 [3.5–5.8] log_10_ U/mL. HDV was undetectable in 4 cases and below LLoQ in 3 additional patients at week 15 (Fig. 1).

Following monotherapy, patients underwent combination therapy with REP 2139-Ca and pegIFN. A total of 9 patients were HDV RNA negative at the end of the full treatment course and 7 of these 9 patients remained HDV RNA negative 2.5 years later^8,12^.

**Figure 2:**
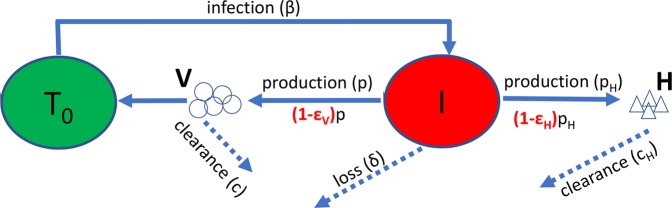
A schematic description of the model (Eq.1). Target cells, *T*_*0*_, are infected with rate β, and become infectious cells *I*. Infectious cells loss at a rate 𝛿, and produce virions, *V*, at rate *p*. After treatment the production rate of virions is reduced by a factor (1-ε_v_). Virions are cleared at rate c. Infectious cells also produce HBsAg,* H*, at rate P_H_. This rate is reduced by a factor (1-ε_H_) after treatment. HBsAg is cleared at rate c_H_. As was done previously^13^, we assume that *T*_*0*_ was constant during the 15 weeks of treatment at its pre-treatment steady-state value.

Overall, the kinetic analysis suggests that: (i) there was no association between changes in HBV DNA and HBsAg levels or between HBV DNA and HDV RNA levels, (ii) anti-HBs sero-conversion occurred in half of responding patients and took place several weeks after HBsAg and HDV started to decline from pre-treatment levels and typically occurred towards the end of treatment, (iii) reminiscent of our previous findings in mono-infected HBV patients^10^, ALT elevations (with the exception of patient 1) did not correlate with changes in HBsAg or HDV levels, and (iv) after apparently similar delays HBsAg and HDV kinetics were highly correlated. Thus, we chose to model only the kinetics of HDV RNA and HBsAg in Eq. 1 (Fig. 2).

### Modeling results

**Figure 3:**
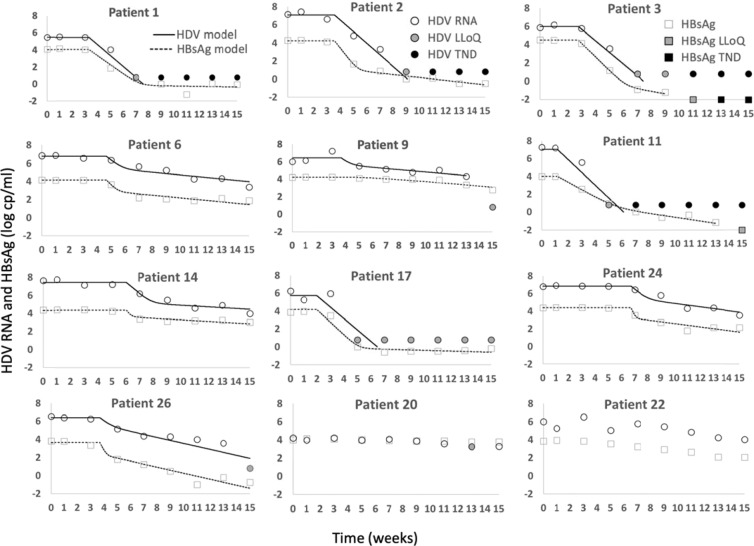
Model calibration (curves) with each patient’s HDV RNA and HBsAg kinetic data (symbols) during 15-week REP 2139-Ca monotherapy. Black filled markers represent values below TND (target not detected) and gray filled markers represent values below LLoQ (lower limit of quantification).

The model (Fig. 2 and Eq. 1) reproduces well the HDV RNA and HBsAg kinetics in the 10 responding patients (Fig. 3) and provides estimates of unknown model parameters (Table 2). Modeling calibration with measured data estimates median baseline HDV RNA *V*_0_ of 6.7 [6.1–7.0] log_10_ U/mL and median baseline HBsAg *H*_0_ of 4.2 [4.1–4.3] log_10_ IU/mL. The median time delay before blockage of HBsAg and HDV RNA production *t*_*b*_ was 25.3 [20.3–32.8] days. The median clearance rate of HBsAg c_H _, was found to be 0.53 [0.38–0.79] days^−1^ corresponding to a HBsAg t_1/2_ = 1.3 days. The estimated HBsAg t_1/2_ implies a median production and clearance of 10^8^ [10^7.7^–10^8.3^] copies/day. The median efficacy of blocking HBsAg production *ε*_*H*_ was 0.982 [0.945–0.999] and the median efficacy of blocking HDV RNA production *ε*_*V*_ was found to be 0.997 [0.959–0.998]. The median estimated loss rate of infected cells *δ*, was 0.052 [0.035–0.074] cells/day. We note that model fits for Pt 9, who was a border-line responder were included in our analysis of the median and IQR, however as these are nonparametric measures, removing this Pt 9 from the analysis had a minimal effect on these values.

**Table 1:** Patient baseline characteristics.

Pt*	Age (Years)	Sex	ALT (U/L)	AST (U/L)	Hepatic stiffness (kPa)	HBsAg (IU/mL)	HBV DNA (IU/mL)	HBcrAg (log U/mL)	HDV RNA (U/mL)	Duration of HDV infection before treatment
1	33	F	188	160	8.4	13988	<10	<LLoD	3.94 × 10^5^	1 year, 5 months
2	29	F	98	64	7.7	27264	<10	<LLoD	4.71 × 10^7^	3 years, 6 months
3	40	M	53	36	14.8	28261	<10	<LLoD	6.97 × 10^5^	18 years
6	37	M	95	54	6.8	17511	726	4.1	5.49 × 10^6^	12 years
9	22	M	85	55	12	16426	104	4.4	2.11 × 10^5^	4 years, 7 months
11	35	M	200	85	9.6	12382	<10	3.2	1.21 × 10^7^	9 years
14	32	M	143	64	11.6	20869	<10	<LLoD	2.30 × 10^7^	6 years, 1 month
17	34	M	62	44	9.5	8314	350	<LLoD	1.69 × 10^6^	10 months
20	44	F	29	27	8.8	13430	<10	4.5	2.74 × 10^4^	12 years
22	36	M	101	78	11.9	7836	16	5	1.09 × 10^6^	1 year, 6 months
24	39	M	160	88	7.8	20473	<10	2.8	1.89 × 10^6^	4 years, 10 months
26	39	M	85	61	30.7	5854	256	4.5	3.76 × 10^6^	9 years
Median	36	-	96	62	9.6	15207	<10	3	1.79 × 10^6^	5 years 6 months

All patients were infected with hepatitis D virus (HDV) genotype 1, negative for hepatitis B e antigen (HBeAg), and positive for anti-HBe. *, patient number as previously reported;^8^ TND, target not detected; LLoQ, lower limit of quantification; LLoD, lower limit of detection; ALT, alanine aminotransferase; AST, aspartate aminotransferase; HBsAg, hepatitis B surface antigen; HBcrAg, hepatitis B core-related antigen.

We did not find any association between the individual fit parameter values *t*_*b*_, *ε*_*H*_, *ε*_*V*_, and *δ* and baseline characteristics including duration of infection, ALT, gender, liver stiffness, *V*_0_, and *H*_0_. We found that *p* > 0.1 for all tested associations. In particular, the lack of association with ALT further justifies excluding ALT dynamics from our model.

## Discussion

In the current study we estimated viral and host kinetic parameters under REP 2139-Ca monotherapy using a model modified from analysis of HDV replication under pegIFN monotherapy^13^. The pre-treatment median and interquartile range for HDV RNA (V_0_ = 6.6 [6.1–7.0] U/mL) were similar to previously studied pegIFN-treated patients (V_0_ = 7.1 [6.2–7.4] U/mL). Likewise, pre-treatment HBsAg in the current study (H_0_ = 4.2 [4.1–4.3] IU/mL) was similar to previous pegIFN-treated patients (H_0_ = 3.9 [3.2–4.1] IU/mL). However, under pegIFN treatment, there was a relatively long delay between the initial decrease in HDV viral load (i.e. after 8.5 [5.3–14.7] days) compared to when the decrease in HBsAg was observed (i.e. after 25.3 [20.3–32.7] days). In contrast, under REP 2139-Ca treatment, our model predicts that both HDV RNA and HBsAg declined after 25.3 [20.3–32.7] days. The HDV blocking efficacy for pegIFN was estimated at 96.2% [93.0–99.8] whereas the corresponding blocking efficacy under REP 2139-Ca was 99.7% (95.9–99.8). Of note, the loss rate of infected cells for pegIFN treatment, δ = 0.0051 [0.0015–0.035] days^−1^, was significantly lower than the loss rate estimated here of δ = 0.052 [0.035-0.073] days^−1^. The reason for this difference in loss rates is unclear, particularly as REP 2139-Ca has not previously been shown to increase loss of infected cells. This difference could be the result of an immune response, although, thus far REP 2139 has not been shown to augment the host immune response to HDV^8,10,11^. Unfortunately, the effects of NAPs observed in humans were not reproduced in rodent models^14^, so alternative methods are needed to determine whether NAPs do affect the immune system and then models could be developed to incorporate such a response.

The estimation of HBsAg turnover based on pegIFN inhibition kinetics is confounded by the multiple antiviral mechanisms of pegIFN, which not only affects viral replication but alters immune function^15,16^. Additionally, we previously showed^13^ that under pegIFN it is not feasible to estimate HBsAg turnover because decreases in HBsAg occurred only during the second phase decrease of HDV RNA, which corresponds to cell death/loss. In contrast, REP 2139-Ca targets HBsAg SVP assembly and secretion^17^, which is the source of almost all circulating HBsAg, allowing for a direct assessment of HBsAg turnover. The estimated half-life of HBsAg (1.3 days) under REP 2139-Ca is strikingly short compared to the half-life of 38 days estimated under lamivudine^18^ and approximately 7-fold shorter than estimated with deuterated HBsAg^19^ suggesting that the turnover of HBV SVP may be more rapid in these patients than estimated in previous studies. However, both studies only examined a small subset of patients and the estimates are within an order of magnitude. The decline in circulating HBsAg is likely driven at least in part by a reduction in HBsAg production/secretion^20,21^. Nonetheless it is also possible that NAPs may increase HBsAg clearance through some yet unknown mechanism. The previous studies investigated HBV mono-infections, whereas the current patients were HBV/HDV coinfected, raising the possibility that co-infections behave differently, and this could perhaps lead to the observed differences.

We also note that our model does not include intracellular dynamics. The identified mechanism of action of REP 2139-Ca consisting of blocking SVP assembly and production with an expected corresponding blockage of HDV production (which is dependent on SVP morphogenesis) is consistent with the patient data. However, the data do not eliminate the possibility of additional modes of action. Preliminary analysis of HDV protein interaction suggests that REP 2139 binds to the small and large forms of HDAg *in vitro*^22^ similar to other sequence independent oligonucleotide interactions with HDAg^23^. These interactions could potentially result in a direct antiviral effect against HDV. However, more frequent sampling in additional patients is needed to separate the onset of blocking HBsAg and HDV production and investigate this possible mechanism using a multiscale modeling approach. Further studies are needed to validate the model presented here and to determine how different concentrations of NAPs relate to the mode of action and effectivity of treatment. Such studies also should investigate changes in HBsAg isoforms during NAP therapy and the effects of NAPs on the release of HBV SVP, Dane particles / HBV filaments and HDV virions.

In conclusion, by simultaneously modeling HBsAg and HDV kinetic data under REP 2139-Ca monotherapy, we estimated for the first time the efficacy of REP 2139-Ca in blocking HBsAg and HDV production, as well as the serum HBsAg half-life and the loss/death rate of HDV-infected cells. These analyses demonstrate a potent effect of REP 2139-Ca against HBsAg and HDV and also seem to indicate that the turnover of HBsAg SVP in the serum may be faster than previously estimated.

## Methods

### Patients

The study population consisted of 12 treatment-naive, HBeAg-negative, HDV RNA positive participants with serum HBsAg titers >1000 IU/mL who were treated with REP 2139-Ca (the calcium chelate complex formulation of REP 2139) for 15 weeks in the phase IIA REP 301 clinical trial^8^. Baseline participant characteristics were as previously described^8^ (Table 1). All methods were carried out in accordance with the Helsinki declaration and the National Ethics Committee and National Medicines Agency of the Republic of Moldova. All patients provided written, informed consent prior to treatment.

**Table 2:** Best model fit results- mean ± standard deviation.

Patient no. as in^8^	Pre-treatment HDV RNA	Pre-treatment HBsAg	Delay before blocking production	HBsAg Clearance rate	Drug Efficacy in Blocking HDV production	Drug Efficacy in Blocking HBsAg	Loss rate of infected cells	HBsAg production ** (IU/day)
	*V*_0_ (log(U) /mL)	*H*_*0*_ (log(IU) /mL)	*t*_*b*_ days	*c*_*H*_ days^−1^	*ε*_*V*_	*ε*_*H*_	*δ* days^−1^	
1	5.5 ± 0.2	4.1 ± 0.2	23.2 ± 3.4	0.35 ± 0.057	0.994^#^	0.999 ± 0.0001	0.008 ± 0.026	10^7.7^
2	7.1 ± 0.1	4.2 ± 0.1	25.0 ± 1.1	0.62 ± 0.099	0.999^#^	0.999 ± 0.0005	0.053 ± 0.012	10^8.2^
3	6.0 ± 0.1	4.5 ± 0.1	19.4 ± 0.6	0.50 ± 0.065	0.998^#^	0.999 ± 3e-5	0.090 ± 0.021	10^8.3^
6	6.7 ± 0.2	4.1 ± 0.2	32.7 ± 1.7	0.57 ± 0.387	0.957 ± 0.025	0.949 ± 0.0299	0.051 ± 0.012	10^8.0^
9*	6.5 ± 0.1	4.2 ± 0.1	25.6 ± 9.3	0.08 ± 0.140	0.867 ± 0.147	0.0030^@^	0.052 ± 0.028	10^7.3^
11^^^	7.0 ± 0.3	4.0 ± 0.3	8.3 ± 0.4	0.28 ± 0.064	0.999^#^	0.999 ± 0.0025	0.074 ± 0.025	10^7.6^
14	7.4 ± 0.1	4.3 ± 0.1	42.1 ± 1.5	0.79 ± 0.341	0.994 ± 0.003	0.827 ± 0.0921	0.029 ± 0.015	10^8.3^
17	5.7 ± 0.4	4.2 ± 0.4	13.9 ± 3.8	0.46 ± 0.067	0.997^#^	0.999 ± 0.0001	0.014 ± 0.030	10^8.0^
24	6.9 ± 0.1	4.4 ± 0.1	46.9 ± 1.6	1.17 ± 1.045	0.966 ± 0.021	0.942 ± 0.0365	0.067 ± 0.016	10^8.6^
26	6.4 ± 0.3	3.6 ± 0.3	25.8 ± 14.6	1.00 ± 1.122	0.905 ± 0.237	0.966 ± 0.0810	0.125 ± 0.062	10^7.8^
**Median (**IQR**)**	6.68 (6.1–7.0)	4.2 (4.1–4.3)	25.3 (20.3–32.7)	0.53 (0.38–0.79)	0.994 (0.959–0.998)	0.982 (0.945–0.999)	0.052 (0.035–0.074)	10^8.0^ (10^7.7^–10^8.3^)

*, fitted until week 13; ^@^, Range not provided due to high uncertainty; ^, Due to fitting constraints, *c* was set to *0.47* days^−1^; ^#^, Minimal estimate since HDV dropped below LLoQ or TND during the first phase of HDV decline; **, As described in Methods; IQR, interquartile range.

### HDV RNA, HBV DNA, anti-HBs, HBsAg, and ALT measurements

Serum HDV RNA (Robogene MK I), HBV DNA (Abbott Realtime) and HBsAg levels (Abbott Architect quantitative) were measured every two weeks. For HDV, the lower limit of quantification (LLoQ) was 3.26 log U/mL, for HBsAg, LLoQ = −1.3 log IU/mL and for HBV DNA, LLoQ=1 log IU/mL. For anti-HBs the architect LLoQ is 2mIU/mL and the current convention, which we followed, is to define seroconversion as a titer> 10mIU/mL^24^. The upper limit of normal for ALT was 50 U/L.

## Mathematical modeling

### Modeling

Previous *in vitro* studies demonstrated that REP 2139 blocks the assembly of HBV subviral particles (SVPs), simultaneously lowering intracellular HBsAg and blocking HBsAg secretion from SVPs^17^. This effect is driven by a post entry mechanism as REP 2139 does not block entry of HBV or HDV into the host cell^25^. Therefore, we modified our previous dual-mathematical model of HDV RNA and HBsAg dynamics under pegIFN therapy^13^ by adding a possible effect of REP 2139 in blocking both HDV and HBsAg production (Eq. 1 and Fig. 2). The modified model includes the following parameters: *I* = productively HDV infected cells, *V* = HDV RNA, *H* = HBsAg, and *T*_0_ = the number of target/susceptible cells (i.e., HBsAg-producing cells). *V*, infects *T*_0_ with constant rate β, generating infected cells, *I*. Parameter *δ* is the loss rate of HDV-infected cells, *p* is the production rate of virions, *c* is the clearance rate constant of virions, *p*_*H*_ is the production rate of HBsAg, and *c*_*H*_ is the clearance rate constant of HBsAg. Treatment is assumed to begin blocking HDV and HBsAg production after time t_b_, with efficacy *ε*_*V*_ *and ε*_*H*_, respectively. Model equations are:

1 $$\begin{array}{c}\frac{d}{dt}(I)=\beta V{T}_{0}-\delta I\\ \frac{d}{dt}(V)=(1-{\varepsilon }_{V})pI-cV\\ \frac{d}{dt}(H)=(1-{\varepsilon }_{H}){p}_{H}I-{c}_{H}H\end{array}$$

### Parameter estimations

To limit the number of unknown model parameters, we fixed *β* =  10^−7^ mL. virions^−1^·days^−1^ and *p* *=* 10 virions·days^−1^ as previously done^13^. The values of *p*_*H*_, and *I*_0_ were set based on the initial steady-state condition leading to ${I}_{0}=c{V}_{0}/p$; pH = c_H_H_0_/I_0_; and ${T}_{0}=\delta {I}_{0}/\beta {V}_{0}$. The viral clearance rate constant was fixed to *c = 0.42* days^−1^ based on our previous estimates^26^. The remaining parameters (V_0_, H_0_, *t*_*b*_*, c*_*H*_, *ε*_*V*_*, ε*_*H*_*, δ*) were estimated for each patient according to the viral kinetics. Data points up to and including the first time HDV and HBsAg are below the LLoQ or target not detected (TND) were included in the fit. Every included data point had equal weight in the fitting based on minimizing least-squares. We used Python 3.7 and Scipy Version 1.0 to estimate the parameter values.

### HBsAg production rate

Having stable levels of HBsAg implies production and clearance are in balance before treatment. Therefore, from Eq. 1 the production rate of serum HBsAg before treatment initiation must equal the HBsAg clearance rate c_H_H_0_. Assuming that the total body fluid volume, F, was 13,360 mL for body weight of 70 kg, as in our previous study^13^ we estimated the total HBsAg production in each patient before treatment initiation by the product F·c_H_·H_0_.

### Statistical analysis

Non-parametric Spearman and Mann-Whitney U Tests were performed using Python version 3.7 and Scipy version 1.3. In all cases P ≤ 0.05 was considered significant.
